# Tannin-Tolerant *Saccharomyces cerevisiae* Isolated from Traditional Fermented Tea Leaf (Miang) and Application in Fruit Wine Fermentation Using Longan Juice Mixed with Seed Extract as Substrate

**DOI:** 10.3390/foods13091335

**Published:** 2024-04-26

**Authors:** Somsay Phovisay, Pratthana Kodchasee, Aliyu Dantani Abdullahi, Nang Nwet Noon Kham, Kridsada Unban, Apinun Kanpiengjai, Chalermpong Saenjum, Kalidas Shetty, Chartchai Khanongnuch

**Affiliations:** 1 Multidisciplinary School, Chiang Mai University, Muang, Chiang Mai 50200, Thailand; somsay2009@hotmail.com (S.P.); ichineung@gmail.com (P.K.); abdullahialiyud.ada@gmail.com (A.D.A.); nwenoonkham@gmail.com (N.N.N.K.); 2 Department of Food Science and Technology, Faculty of Agriculture and Forest Resource, Souphanouvong University, Luang Prabang 06000, Laos; 3 Division of Food Science and Technology, Faculty of Agro-Industry, Chiang Mai University, Muang, Chiang Mai 50100, Thailand; kridsada.u@cmu.ac.th; 4 Research Center for Multidisciplinary Approaches to Miang, Multidisciplinary Research Institute (MDRI), Chiang Mai University, Chiang Mai 50200, Thailand; apinun.k@cmu.ac.th; 5 Department of Chemistry, Faculty of Science, Chiang Mai University, Huay Kaew Rd., Muang, Chiang Mai 50200, Thailand; 6 Faculty of Pharmacy, Chiang Mai University, Muang, Chiang Mai 50100, Thailand; chalermpong.s@cmu.ac.th; 7 Global Institute of Food Security and International Agriculture (GIFSIA), Department of Plant Sciences, North Dakota State University, Fargo, ND 58108, USA; kalidas.shetty@ndsu.edu; 8 Department of Biology, Faculty of Science, Chiang Mai University, Huay Kaew Rd., Muang, Chiang Mai 50200, Thailand; 9 Research Center of Microbial Diversity and Sustainable Utilization, Chiang Mai University, Huay Kaew Rd., Chiang Mai 50200, Thailand

**Keywords:** *Saccharomyces cerevisiae*, tannin tolerance, fermented tea, longan fruit wine

## Abstract

This study focused on isolating tannin-tolerant yeasts from Miang, a fermented tea leaf product collected from northern Laos PDR, and investigating related food applications. From 43 Miang samples, six yeast isolates capable of ethanol production were obtained, with five isolates showing growth on YPD agar containing 4% (*w*/*v*) tannic acid. Molecular identification revealed three isolates as *Saccharomyces cerevisiae* (B5-1, B5-2, and C6-3), along with *Candida tropicalis* and *Kazachstania humilis*. Due to safety considerations, only *Saccharomyces* spp. were selected for further tannic acid tolerance study to advance food applications. Tannic acid at 1% (*w*/*v*) significantly influenced ethanol fermentation in all *S. cerevisiae* isolates. Notably, B5-2 and C6-3 showed high ethanol fermentation efficiency (2.5% *w*/*v*), while others were strongly inhibited. The application of tannin-tolerant yeasts in longan fruit wine (LFW) fermentation with longan seed extract (LSE) supplementation as a source of tannin revealed that C6-3 had the best efficacy for LFW fermentation. C6-3 showed promising efficacy, particularly with LSE supplementation, enhancing phenolic compounds, antioxidant activity, and inhibiting α-glucosidase activity, indicating potential antidiabetic properties. These findings underscore the potential of tannin-tolerant *S. cerevisiae* C6-3 for fermenting beverages from tannin-rich substrates like LSE, with implications for functional foods and nutraceuticals promoting health benefits.

## 1. Introduction

*Saccharomyces cerevisiae* has been widely used for food applications such as winemaking, baking, and brewing [1]. In particular, various strains of *S. cerevisiae* play a crucial role in the qualitative properties of alcoholic beverages by enhancing the nutritional composition and sensory qualities of the final product [2,3]. Fermented tea leaf, or Miang, is a traditional food of northwestern Laos PDR. The steamed tea leaves (*Camellia sinensis* var. *assamica*) are fermented by natural microbial succession to transform a variety of chemical components in tea leaves and enhance organic acid qualities, flavor, and taste by natural microflora, which provide the final product with qualitative edible characteristics [4]. During Miang fermentation, a high degree of microbial diversity such as lactic acid bacteria [5,6], yeast [7], and *Bacillus* spp. [8] has been reported, and the majority of these microbes displayed tannin-tolerant characteristics. Moreover, Tannin-tolerant *Saccharomyces cerevisiae*, isolated from Miang in Northern Thailand, has been identified to possess significant potential for bioconversion of high-tannin substrates, making it suitable to produce fuel ethanol or functional alcoholic beverages. Previous research has highlighted the significant advantages of tannin-tolerant *S. cerevisiae* strain ML1-2 isolated from a Miang sample collected from Northern Thailand in the fermentation of Java plum musts mixed with ground seed. *S. cerevisiae* ML1-2 showed significant advantages in growth and enhanced contents of ethanol, polyphenols, tannin, and flavonoids compared to a reference strain, *S. cerevisiae* TISTR 5088 [9]. Additionally, the use of tannin-tolerant non-*Saccharomyces* yeast isolated from Miang, such as *Wikerhamomyces anomalus* and *Cyberlindnera rhodanensis,* from the fermentation of Miang residual broth provided different volatile organic compound profiles, with elevated levels of ester groups like ethyl acetate and isoamyl acetate [10].

Tannin is a complex polyphenol that occurs widely in nature and is mainly found in various parts of plants, including leaves, fruits, seeds, and barks [11]. Phenolic compounds in tannins are considered enzyme inhibitors for health benefits such as anti-hypertension, anti-type II diabetes, anti-obesity, and antioxidant activity [12,13]. However, high tannin levels are proven to produce a marked inhibitory effect on the growth of yeast in alcoholic fermentation [14,15]. The mechanisms of inhibition by tannins against microbes have been suggested to consist of iron chelation, inhibition of cell wall synthesis, disruption of the cell membrane, and fatty acid biosynthetic pathways [16]. The minimum inhibitory concentration (MIC) and the minimum bactericidal concentration (MBC) of antimicrobial activity against *S. cerevisiae* were previously reported to be 12.5 and 25.0 mg/mL, respectively [17].

Longan (*Dimocarpus longan* Lour.) is one of the major fruits in north Thailand. This fruit is processed commercially into dried, canned, and frozen products to extend its shelf-life and exports. During the harvest season, there are high quantities of longan fruits in the market, causing a price reduction (Office of Agricultural Economics, Ministry of Agriculture and Cooperatives, Thailand, 2019). Likewise, a large amount of longan seeds is normally accumulated as by-products during longan processing, which contains an abundance of bioactive compounds with potential health benefits. Previous studies in the literature reported that longan seed contained nutritional phytochemicals consisting of chebulagic acid, isomallotinic acid, geraniin, brevifolin, butanoic acid, caffeic acid, and flavogallonic acid [18,19]. Wang et al. [20] also reported that the condensed tannin in longan seed was found to be up to 69.53 ± 1.99 mg/g (DW). The longan seed extract exhibits excellent antioxidant activity due to its rich content of bioactive compounds, including phenolic acids and polysaccharides. Therefore, it holds great potential as a readily available source of natural antioxidants for both the food and pharmaceutical industries [21,22]. In addition, LSE was proposed as a source of potential phytochemicals for promoting anti-angiogenesis activity and assisting memory [23,24,25] and has also been reported for its antimicrobial activity against various pathogenic bacteria, including *Streptococcus aureus*, *Escherichia coli*, *Pseudomonas aeruginosa*, and *Salmonella* [20,26]. In order to add value to waste byproducts from abandoned longan fruit and longan seed waste generated during the longan harvesting season, this research aimed to process the longan fruit and its derived waste into a healthy drink containing health-beneficial compounds. This advances the effort to utilize the microbial resources from Miang to create value-added products from agricultural byproducts like longan seeds in the development of functional beverages. The fermentation process of agricultural byproducts like longan seeds by tannin-tolerant *S. cerevisiae* strains is also an attractive approach to the development of functional beverages with health benefits. This research underscored the potential of tannin-tolerant *S. cerevisiae* strains to improve the functionality of fermented beverages, aligning with broader efforts to advance food innovations.

This specific study describes the isolation of tannin-tolerant *S. cerevisiae* from Miang samples collected from Lao PDR and utilizing the selected yeast for investigating LFW fermentation using seed extract as a tannin source to evaluate their fermentation efficiency and potentially enhance health benefits.

## 2. Materials and Methods

### 2.1. Raw Materials and Chemicals

Miang samples were collected from local markets and Miang production areas in the northwestern region of Laos (Xayaboury, Laos PDR). Longan fruit and seed were purchased from a local longan farm (Chiang Mai, Thailand). Tannic acid (Gallotannin) was purchased from LOBA Chemie (Mumbai, India). Chloramphenicol was purchased from Bio Basic Inc. (Markham, ON, Canada), and ethyl alcohol (95% food grade) was purchased from Union Chemical and Equipment CO. LTD (Bangkok, Thailand). The reference strain (*S. cerevisiae,* TISTR 5088) was obtained from the Thailand Institute of Scientific and Technology Research (TISTR, Pathum Thani, Thailand).

### 2.2. Sampling Sites and Sample Collection

**Figure 1:**
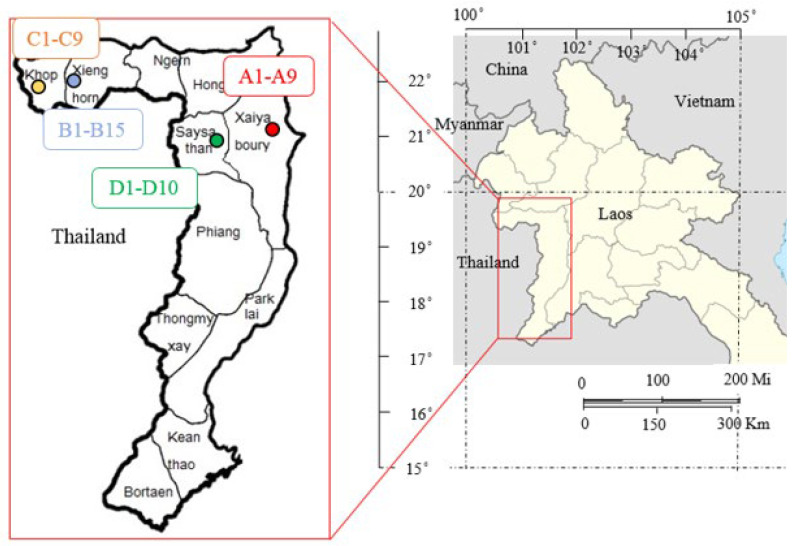
Map of Miang sampling location in Xaiyabury province of Laos.

A total of 43 Miang samples were collected from 4 districts in Xayaboury province (Laos PDR), including Xayaboury, Xienghorn, Khop, and Saysathan (Table 1), and sampling sites were plotted on a map (Figure 1). These places are well-known regions of Miang cultivation and Miang fermentation aligned with the local customs of the villagers who established the settlement in northwest Laos. Each sample was collected aseptically in a plastic bag, maintained in an ice box, and delivered for keeping at 4 °C until analyzed.

### 2.3. Isolation and Screening of Tannin-Tolerant S. cerevisiae from Miang

#### 2.3.1. Isolation of Yeast

A total of 20 g of each collected Miang sample was mixed with 100 mL of sterile normal saline (0.85% *w*/*v* NaCl). Subsequently, 1 mL of the aqueous solution was transferred into 10 mL of YPD broth (containing 10 g/L yeast extract, 20 g/L peptone, and 20 g/L glucose), supplemented with 100 ppm of chloramphenicol (an antibacterial agent). A Durham tube was placed inside each YPD tube to detect gas production. All cultures were incubated at 30 °C for 2–5 days. The culture broth of samples that showed a positive result for gas production was spread onto YPD agar to obtain single yeast colonies. Yeast morphological characteristics were observed and used to separate colonies based on appearance and cellular morphology under a microscope. The pure cultures were preserved at −20 °C in YPD broth containing 15% (*v*/*v*) glycerol.

#### 2.3.2. Screening of Gas Formation and Ethanol Production

The ability to produce gas was initially used to isolate ethanol-fermenting yeast based on the principles of ethanol fermentation. During ethanol fermentation, fermentable sugars are converted into ethanol and carbon dioxide through microbial activity (C_6_H_12_O_6_ → C_2_H_5_OH + CO_2_). Each individual culture that tested positive for gas production was anaerobically examined and categorized based on the formation of gas (CO_2_) in the Durham tube as well as foam production during the shaking step. The cultures that were confirmed to exhibit gas production were then subjected to preliminary evaluation for ethanol fermentation in YPD broth using an ebulliometer.

#### 2.3.3. Tannin Tolerance Assessment of Ethanol-Producing Yeast

The potential of yeast for tannin tolerance was determined using a modified method, according to Kanpiengjai et al. [7]. Briefly, the tannic acid (TA) solution (adjusting a pH of 7.0 with NaOH) and YPD agar were prepared individually before being autoclaved at 121 °C for 15 min. The sterile tannin solution and melted YPD agar were mixed aseptically to final concentrations (*w*/*v*) of l, 2, 3, and 4% before preparing tannic agar plates. A single colony previously confirmed based on gas formation and ethanol production was selected and streaked on varying tannic agar to indicate tannin-tolerant ability by the growth of each isolate after incubation for 2 days.

### 2.4. Molecular Identification of Tannin-Tolerant S. cerevisiae Isolated from Miang

The DNA of yeast isolates from Miang, which had the ability to produce ethanol, and have tannin tolerance, was identified by sequence analysis of the D1/D2 domain of the LSU rRNA gene. Each isolate was grown in YPD broth at 30 °C for 24 h, then the cells were harvested by centrifugation (Velocity 14R, Dynamica Scientific Ltd., Livingston, UK) at 14,000× *g* for 1 min at 4 °C and resuspended in 480 μL of 50 mM ethylenediaminetetraacetic acid (EDTA). A volume (40 μL) of a 50 mg/mL solution of lysozyme (Bio Basic Inc., Markham, ON, Canada) was added to the cell suspension. The genomic DNA was extracted using a Wizard Genomic DNA purification kit (Promega, Madison, WI, USA) following the manufacturer’s protocol for genomic DNA from yeast. Then, extracted DNA was amplified using universal primers NL1 (5′-GCATATCAATAAGCGGAGGAAAAG-3′) and NL4 (5′-GGTCCGTGTTTCAAGACGG-3′) [27]. The polymerase chain reaction was undertaken in an MG-96 MyGene^TM^ Thermal Cycler (LongGene Scientific Inc., Changzhou, China). The PCR products were purified and sequenced by a sequencing service provider (1st BASE Pte Ltd., Singapore). The pairwise sequence alignment of the D1/D2 sequences was compared to other genes in the GenBank databases, and the phylogenetic tree was established based on the neighbor-joining method by MEGA version 4.0 software.

### 2.5. Assessment of Ethanol Production Capability in Tannin-Enriched Medium

The performance of three *S. cerevisiae* yeast strains isolated from Miang and a reference strain (*S. cerevisiae* TISTR 5088) was comprehensively assessed in the presence of varying concentrations of TA (0%, 1%, 2.5%, and 5%). The assessment included the measurement of several parameters, including viable cell count, pH monitoring, sugar utilization, and ethanol production.

#### 2.5.1. Viable Cell Count and pH Monitoring

A single colony from each yeast strain was cultured in YPD broth for 16–18 h at 30 °C with continuous agitation (150 rpm). A seed culture (10% *v*/*v*) was then inoculated into the different TA-containing media, each with a total volume of 200 mL. These cultures were incubated statically at 30 °C under airlock conditions. The viable cell counts and pH levels were closely monitored at specific intervals, including 0, 6, 12, and 24 h. This allowed the yeast’s response to TA and its impact on cell growth in the surrounding environment to be assessed.

#### 2.5.2. Evaluation of Sugar Utilization and Ethanol Production

In parallel with the growth assessment, a comprehensive analysis of sugar utilization and ethanol production was conducted. Yeast cultures were sampled at 6 h intervals and subsequently centrifuged at 5000 rpm for 10 min at 4 °C. The resulting cell-free supernatant was subjected to analysis using high-performance liquid chromatography (HPLC). The HPLC analysis employed a refractive index detector, an autosampler, an Aminex HPX-87H Column (300 × 7.8 mm, Bio-Rad, Hercules, CA, USA) as the stationary phase, and a mobile phase consisting of 0.005N sulfuric acid. HPLC analysis was conducted at a flow rate of 0.75 mL/min and a temperature of 40 °C.

The ethanol productivity (g/L·h) was calculated as the ratio of ethanol concentration (g/L) at the fermentation time (h) (Equation (1)).

(1) $$\mathrm{Productivity}(g/L\cdot h)=\frac{\mathrm{Ethanol}}{\mathrm{Time}}$$

The fermentation efficiency was evaluated according to Equation (2):

(2) $$\mathrm{Efficiency}\;(\%)=\frac{\mathrm{Practical}\;\mathrm{yield}\;\mathrm{of}\;\mathrm{ethanol}}{\mathrm{Theoretical}\;\mathrm{yield}\;}\times 100$$

### 2.6. Application of Tannin-Tolerant S. cerevisiae in Longan Fruit Wine Fermentation Supplemented Seed Extract

#### 2.6.1. Longan Seed Extraction

The performance of three *S. cerevisiae* yeast strains isolated from Miang and a reference strain was evaluated with longan seed extract. The longan seed was dried in a vacuum oven (Binder VD 53, Binder GmbH, Tuttlingen, Germany) and ground into a fine powder using a kitchen grinder. The dried longan seed was extracted using the maceration technique with a modified method [28]. In brief, 100 g of ground longan seed was extracted with 500 mL of 95% ethanol as the solvent. This mixture was shaken continuously on a magnetic stirrer for 4 h, and the extracted solvent was then filtered through Whatman No. 4 filter paper. The residue from the filtered cake underwent three rounds of extraction. Subsequently, all the filtrate was concentrated under vacuum conditions at 50 °C using a rotary evaporator. The semi-dried crude extract was mixed with distilled water at a 1:20 volume ratio and transferred to a separating funnel to obtain the LSE as a source of crude tannin extract, which appeared as a black aqueous phase at the bottom. This solution was stored at 4 °C. Prior to use as a tannin source in LFW fermentation, the LSE solution was analyzed for total polyphenols and total tannin content following the method described by Makkar et al. [29].

#### 2.6.2. Preparation of Longan Fruit Wine Fermentation Supplemented Seed Extract

Fresh longan fruit (E-dor cultivars) was obtained from a local longan farm in Chiang Mai province, Thailand. The longan aril was blended to extract longan juice through a filter cloth. The total soluble solids and pH of the longan juice were adjusted to approximately 14% Brix and 6.00 (using water and NaOH), respectively. The LSE obtained through maceration extraction (containing 92.08 mg GAE/mL of tannin) was added to longan juice as a tannin source at three different levels: 0%, 0.25%, and 0.5% (*w*/*v*). The mixture was then autoclaved at 121 °C for 15 min. The sterile longan juice supplemented with LSE was fermented using three strains of *S. cerevisiae* (B5-2, C6-3) and TISRT5088 (a reference strain). The fermentation was conducted at 30 °C for 9 days. Throughout the fermentation process, samples of the longan juice were collected aseptically to monitor dynamic changes in cell growth and chemical properties.

#### 2.6.3. Analysis of Total Polyphenols, Total Tannin, and Total Flavonoids

The total polyphenolic content (TP) was measured using the Folin–Ciocalteau reagent method as described by Eom et al. [30]. In brief, 200 µL of the sample was added to a test tube containing 200 µL of 2 M Folin–Ciocalteau reagent and vortexed. After the addition of 1.8 mL of deionized water, the mixture was incubated at room temperature for 3 min. Then, 400 µL of 10% (*w*/*v*) sodium carbonate was added and vortexed. The final volume was adjusted to 4 mL with deionized water, and the mixture was incubated again in the dark at room temperature for 1 h. The absorbance was measured at 725 nm using a UV/VISIBLE spectrophotometer (Metertech SP8001, Taipei, Taiwan). Gallic acid was used as the standard, and the results were expressed as mg of gallic acid equivalents (GAE)/mL of the sample.

The total tannin content (TT) was determined with a modification of the Folin–Ciocalteu reagent method [29]. Tannin was separated from other phenols using polyvinylpolypyrrolidone (PVPP). Briefly, 1 mL of the sample was mixed with 1 mL of 10% (*w*/*v*) PVPP, vortexed, and kept at 4 °C for 15 min. The solution was then centrifuged at 3000 rpm for 10 min, and the supernatant was collected. The total phenolic content of the PVPP-precipitated supernatant was measured with the Folin–Ciocalteu reagent, and tannin content was estimated using the following formula: TT = TP − TP_(PVPP precipitation)_.

The total flavonoid content (TF) was determined by the aluminum chloride colorimetric method. Briefly, 100 µL of 10% (*w*/*v*) aluminum nitrate and 100 µL of 1 M potassium acetate were mixed with 500 µL of the sample. Then, 3.3 mL of 80% (*v*/*v*) methanol was added to the reaction mixture in a test tube, followed by incubation for 40 min. The absorbance of the combination was detected at 415 nm. The absorbance value was then used to estimate the TF by comparing it to a quercetin equivalent (QE)/mL of sample [30].

#### 2.6.4. Antioxidant and α-Glucosidase Inhibitory Activity

The antioxidant activity (AOA) of LFW was assessed by comparing it to an ascorbic acid equivalent. In brief, 4 mL of a 0.15 mM solution of 2,2-diphenyl-1-picrylhydrazyl (DPPH) in an 80% (*v*/*v*) methanol solution were added to 1 mL of diluted LFW, and the mixture was vigorously mixed. After a 30 min incubation in darkness at room temperature, the absorbance was measured at 517 nm. The AOA was expressed as µg of ascorbic acid equivalents (AAE)/mL of sample.

To evaluate α-glucosidase enzyme inhibition, an in vitro assay was conducted [13]. Initially, 50 µL of the sample was mixed with 50 µL of 1.0 M phosphate buffer. Subsequently, 100 µL of α-glucosidase enzyme solution (1.0 U/mL, prepared in phosphate buffer at pH 6.9) was added, and the mixture was incubated for 10 min at room temperature (30 °C). Following this, 50 µL of the substrate (5 mM *p*-nitrophenyl-α-glucopyranoside solution, prepared in the same phosphate buffer) was added at specific intervals and incubated for an additional 5 min. The reaction was stopped by adding 300 µL of 0.1 M sodium carbonate, and absorbance readings were taken at 0 and 5 min after substrate addition at 405 nm using a spectrophotometer (Metertech SP-8001 UV/Visible Spectrophotometer, Metertech Inc., Taipei, Taiwan). The absorbance of the sample was compared to a control containing 50 µL of potassium phosphate-buffered solution instead of the sample, where A represents absorbance. The enzyme inhibition was calculated as a percentage inhibition using the Formula (3):

(3) $$\mathrm{Inhibition}\;(\%)=\frac{\left(Acontrol-Asample\right)}{(Acontrol)\;}\times 100$$

### 2.7. Statistical Analysis

The significant differences in fermentation efficiency and bioactive compounds were analyzed using one-way analysis of variance (ANOVA). A randomized complete block design was employed to assess the statistical significance of these differences through Duncan’s multiple range test (*p* < 0.05). The data were analyzed using SPSS software, version 17.

## 3. Results and Discussion

### 3.1. Isolation of Tannin-Tolerant and Ethanol-Producing Yeast

**Figure 2:**
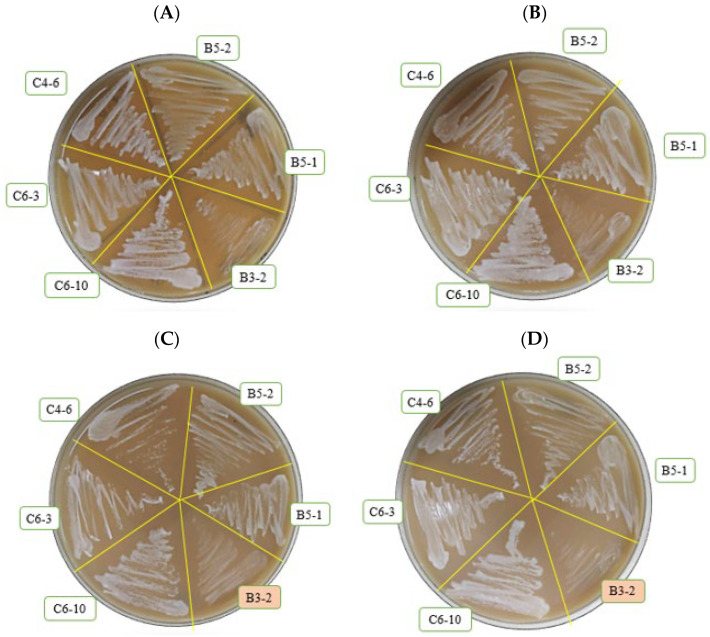
The growth of six selected ethanol-producing yeasts on YM medium supplemented with varied tannic agar concentrations (1–4% *w*/*v*); (**A**) 1%; (**B**) 2%; (**C**) 3%; and (**D**) 4% at 30 °C incubation for 24 h.

A total of 43 Miang samples was collected from household producers and local markets in four regions of northwestern Laos PDR (Table 1 and Figure 1). Preliminary screening involved cultivating these samples in YPD broth to detect gas formation. This screening revealed that 10 out of the 43 samples exhibited gas formation. Subsequently, a total of 35 yeast isolates were obtained from the 10 positive samples for further screening to identify ethanol-producing yeast strains. Among the 35 selected yeast isolates, only six isolates (B3-2, B5-1, B5-2, C4-6, C6-3, and C6-10) were verified for their ability to produce ethanol. The potential for tannin tolerance was assessed and initially screened across varying tannic acid agar concentrations (1–4%, *w*/*v*). It was observed that all six isolates were capable of growth on tannic acid containing agar at concentrations of 1–2%. However, only five isolates (B5-1, B5-2, C4-6, C6-3, and C6-10) displayed growth at higher concentrations, reaching up to 4% TA. In contrast, isolate B3-2 exhibited sensitivity to the inhibitory effects of TA at concentrations ranging from 3% to 4% (*w*/*v*) (Figure 2). The microbial Isolates capable of growing under these conditions are expected to be tannin-tolerant, exhibiting varying degrees of tolerance derived from diverse environmental sources. Previous studies have highlighted the microbial community in Miang fermentation, which includes lactic acid bacteria, yeast, and filamentous fungi, all playing crucial roles in the Miang process. The natural selection of microbial populations with tannin-tolerance capabilities is a fundamental mechanism in fermentation processes [8,31]. In another study, Kham et al. [32] identified groups of yeast isolates from traditional fermented tea capable of growth within a range of 0.5% to 3%. Similarly, Kanpiengjai et al. [7] reported that yeast isolates in Miang demonstrated tannin tolerance characteristics and the presence of tannase-degrading enzymes during Miang fermentation. These findings suggest that ethanol-producing and tannin-tolerant yeast isolated from Miang in response to the high-tannin content of Miang leaves have experienced a natural selection evolution. This process could potentially lead to the development and selection of tannin-tolerant yeast strains within the Miang ecosystem.

### 3.2. Molecular Identification of Tannin-Tolerant Selected Yeasts by rRNA Gene Sequence Analysis

**Figure 3:**
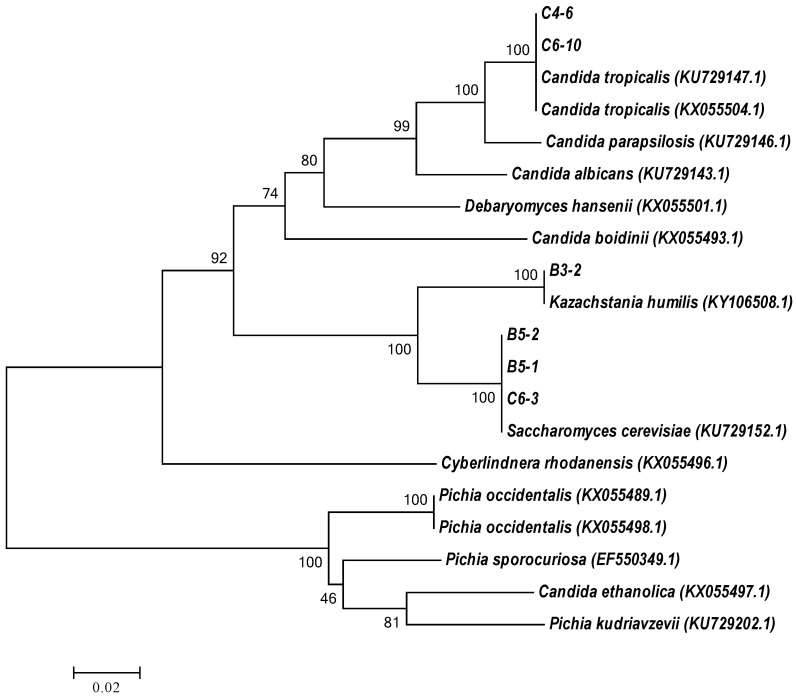
Neighbor-joining tree based on the complete D1/D2 sequence of the LSU rRNA gene.

All six isolates of ethanol-producing and tannin-tolerant yeasts were analyzed phenotypically using sequence analysis of the D1/D2 domain of the LSU rRNA gene to identify their species (Table 2), and the D1/D2 sequence analysis revealed that three isolates, including B5-1, B5-2, and C6-3, were close to *S. cerevisiae* (NG_042623.1) at 99.64–100% similarity. On the other hand, two of the six isolates (C4-6 and C6-10) were closer to *Candida tropicalis*, while isolate B3-2 was close to *Kazachstania humilis*. A phylogenetic tree of ethanol-producing and tannin-tolerant yeasts was constructed based on the neighbor-joining method using the sequences of the D1/D2 domain of the LSU rRNA gene, along with identified yeast species and reference sequences of their closest relatives (Figure 3). The yeast *Candida tropicalis* is considered the second most virulent in terms of clinical importance [33]. Consequently, yeast isolates C4-6 and C6-10 may not be suitable for further applications, particularly in processes related to food. On the other hand, *Kazachstania humilis* is typically found in sourdough and kefir cultures [34]. Therefore, *Kazachstania humilis* B3-2 can possibly be used for further application, especially in food processing; however, more characterizations of this yeast strain, both the special characteristics and safety concerns, are essentially required.

### 3.3. Evaluation of Tannin-Tolerant S. cerevisiae Isolated from Miang on Ethanol Production with Tannin-Enriched Medium

#### 3.3.1. Viable Cell Count and pH of Tannin-Tolerant *S. cerevisiae*

**Figure 4:**
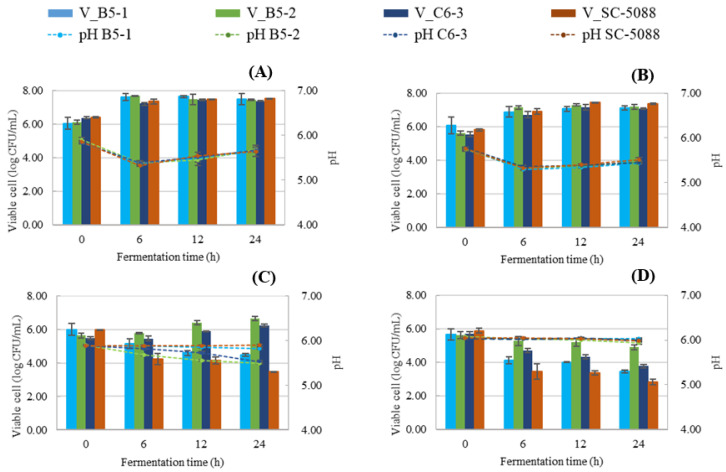
Viable cell count (bar chart) and pH of culture broths from *S. cerevisiae* isolated from Miang and reference strain (*S. cerevisiae* TISTR 5088) statically cultivated at 30 °C in YPD broth containing varied tannic acid concentrations ((**A**) 0%; (**B**) 1%; (**C**) 2.5%, and (**D**) 5%).

Three strains of *S. cerevisiae* (B5-1, B5-2, and C6-3) isolated from Miang and a reference strain (SC-5088) were compared in YPD broth containing TA (tannic acid) at final concentrations of 0%, 1%, 2.5%, and 5% (*w*/*v*). The results indicated initially pH dropped to 5.30–5.40 at 6 h and then slightly increased, reaching 5.44–5.69 at 24 h for all strains cultured in both TA concentrations ranging from 0–1% (*w*/*v*) (Figure 4A,B). In the case of 2.5% (*w*/*v*) TA, the pH of B5-2 and C6-3 steadily decreased from 5.90 to 5.50–5.53 at 24 h, whereas B5-1 and SC-5088 maintained a constant pH value (Figure 4C). Notably, at the highest concentration of 5% (*w*/*v*) TA, the pH value remained unchanged for all strains throughout the fermentation process (Figure 4D). For viable cell count in the absence of TA, all yeast strains exhibited rapid growth, with cell counts ranging from 7.24 to 7.70 log CFU/mL at 6 h (Figure 4A). The addition of 1% (*w*/*v*) TA significantly delayed reproduction in all yeast strains, resulting in lower growth compared to the 0% TA condition. Viable cell counts were recorded at 6.89–7.16 log CFU/mL at 6 h and continued to increase to 7.10–7.38 log CFU/mL at 24 h (Figure 4B). At 2.5% (*w*/*v*) TA, B5-2, and C6-3 were able to grow with a slight increase in cell numbers to 6.65 and 6.23, respectively, while B5-1 and SC-5088 experienced inhibited cell growth that declined gradually during fermentation (Figure 4C). At the critical concentration of 5% (*w*/*v*) TA, all yeast strains exhibited extreme sensitivity and displayed reduced cell counts during cultivation in the presence of 5% (*w*/*v*) TA (Figure 4D). The decrease in pH during *S. cerevisiae* fermentation primarily results from the yeast’s production of organic acids as they metabolize sugars. This change in acidity directly influences yeast cell growth. However, the addition of TA inhibited yeast cell growth due to its strong ability to form chelating complexes with metal ions. This action deprives yeast of essential metal ions, consequently interfering with both yeast growth and metabolism [35,36,37].

#### 3.3.2. Glucose Consumption and Ethanol Production

**Figure 5:**
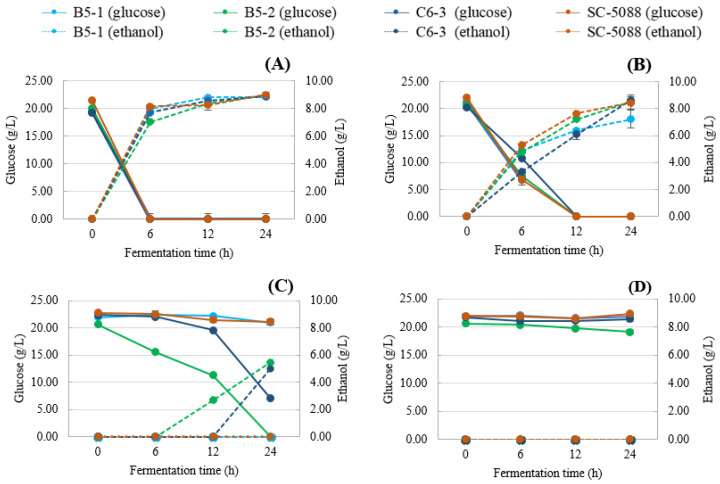
Glucose consumption (linear line) and ethanol production (dash line) of *S. cerevisiae* yeast strains isolated from Miang and the reference strain (*S. cerevisiae* TISTR 5088) at 30 °C cultivation in YPD broth containing tannic acid concentration ((**A**) 0%; (**B**) 1%; (**C**) 2.5% and (**D**) 5%).

In total, four *S. cerevisiae* strains (B5-1, B5-2, C6-3, and SC-5088) were compared for glucose consumption and ethanol production in varied concentrations of tannic acid medium (Figure 5). Overall, all strains were able to convert glucose to ethanol at 0 and 1% (*w*/*v*) TA. The addition of TA at 1% delayed the metabolism associated with productivity. However, at the 24 h mark, three of four strains exhibited the same level of ethanol production (B5-2: 8.51 g/L, C6-3: 8.62 g/L, and SC-5088: 8.43 g/L), except for B5-1, which showed a lower production at 7.22 g/L) (Figure 5B). At 2.5% of TA, ethanol production was disrupted, and only two of four strains revealed a greater potential of ethanol production at 24 h (B5-2: 5.48 g/L and C6-3: 5.01 g/L) in tannin-enriched medium than B5-1 and SC-5088 which were sensitive to 2.5% (*w*/*v*) TA (Figure 5C). In addition, all yeast strains were not able to produce ethanol when cultured at 5% (*w*/*v*) TA (Figure 5D).

The efficacy and productivity of ethanol production by four *S. cerevisiae* strains (B5-1, B5-2, C6-3, and SC-5088) were evaluated for their glucose consumption and ethanol production abilities with varying TA concentrations. The assessment of ethanol production efficacy and productivity post-fermentation analysis at 24 h revealed differences among strains (Table 3). Notably, B5-1 exhibited significantly lower productivity (0.30 g/L·h) compared to the others (B5-2, C6-3, and SC-5088) when exposed to 1% TA. Moreover, productivity was limited at 2.5% TA, with B5-2 and C6-3 producing 0.23 g/L·h and 0.21 g/L·h, respectively. Tannin-free medium yielded the highest efficiency (ranging from 86.80% to 87.86%) for all strains. On the other hand, the addition of 1% TA significantly reduced fermentation efficiency for all strains (B5-1: 74.81%, B5-2: 83.79%, C6-3: 84.39%, and SC-5088: 83.75%), with efficiency remaining in the range of 54.03% to 49.92% when using isolated strains B5-2 and C6-3. These findings indicate that tannins interfere with yeast metabolism and ethanol production through multiple mechanisms, including exerting toxic effects on yeast cells, chelating essential metal ions, disrupting sugar uptake, and ultimately reducing the efficiency of the fermentation process [38,39]. The extent of these effects varies depending on the concentration of tannins in the fermentation medium. Additionally, tannins have been observed to inhibit the activity of the cinnamate decarboxylase in *S. cerevisiae*. This inhibition may be particularly relevant in the context of red wines, where tannins are abundant [40].

### 3.4. Dynamic Changes in Viable Cell Count and Biochemical Property during Longan Fruit Wine Fermentation Supplemented Longan Seed Extract

#### 3.4.1. Viable Cell Count and pH of Tannin-Tolerant *S. cerevisiae*

**Figure 6:**
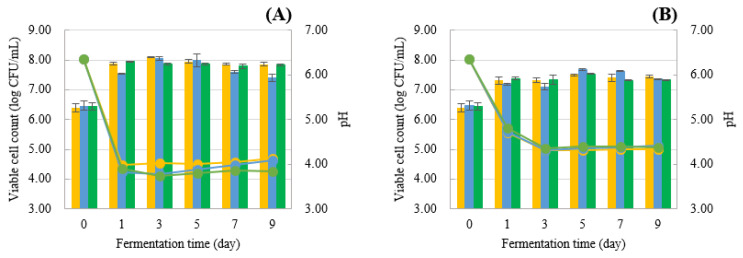

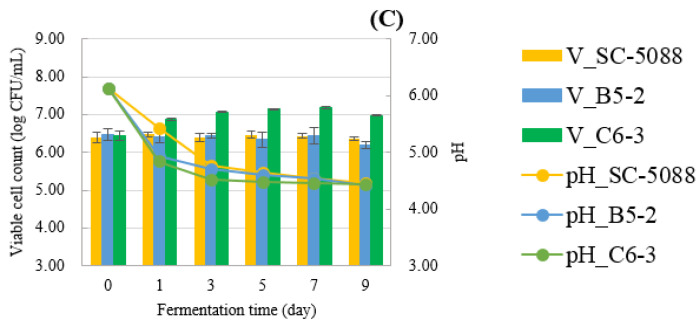
Dynamic changes in the viable cell (bar chart) and pH (linear line) during longan fruit wine fermentation at 30 °C with LSE supplementation ((**A**) 0%; (**B**) 0.25%; (**C**) 0.5%).

The capabilities of selected strains (B5-2, C6-3, and SC-5088) were tested in the fermentation of longan fruit into wine supplemented with various LSEs, and their characteristics were examined. Initially, the pH values (6.00 ± 0.20) of all strains decreased during fermentation. However, the addition of various LSEs resulted in a slight decline in pH. Specifically, the pH levels in the LSE at concentrations of 0%, 0.25%, and 0.5% (*w*/*v*) were observed to be in the range of 3.85–4.13, 4.35–4.43, and 4.42–4.46, respectively, at 9 days (Figure 6A–C). In terms of viable cell counts, all yeast strains exhibited an increase in viable cells when cultured in medium containing 0% and 0.25%, with counts ranging from 7.40–7.86 log CFU/mL and 7.33–7.44 log CFU/mL, respectively. However, in the presence of a higher concentration of LSE 0.5% (*w*/*v*), only one of the three strains (C6–3) showed a gradual and steady increase over 7 days, reaching 7.19 log CFU/mL, followed by a slight decrease to 6.98 log CFU/mL at the end of fermentation. On the other hand, other strains (B5-2 and SC-5088) showed a consistent trend in cell numbers, ranging from 6.40–6.47 log CFU/mL over 7 days, followed by a slight decline to a range of 6.19–6.37 log CFU/mL over 9 days (Figure 6C). At higher concentrations (0.5% *w*/*v*), LSE adversely affected yeast cells, resulting in decreased cell viability and growth, a phenomenon notably observed in strains B5-2 and SC-5088. This impact arises from the potential disruption of yeast cell membranes by the tannin extracts, thereby impeding essential cellular processes and ultimately reducing viable cell counts. Notably, tannin-tolerant yeast strains were identified among different yeast strains (B5-2, C6-3, and SC-5088), with C6-3 demonstrating greater resilience to the adverse effects induced by tannins compared to the other strains.

#### 3.4.2. Sugar Utilization and Ethanol Production

**Figure 7:**
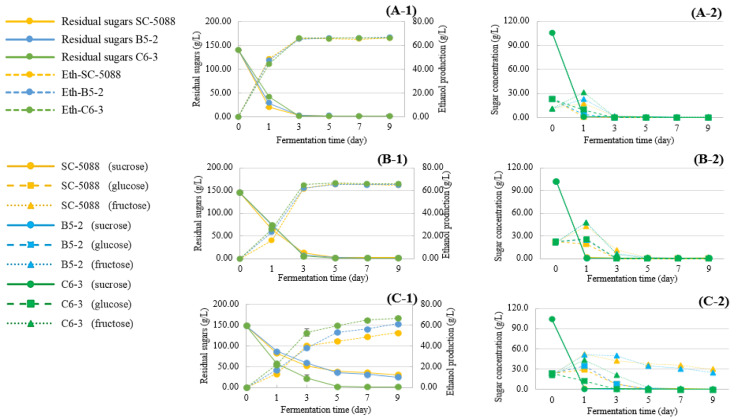
Comparison of sugar consumption and ethanol production by the selected yeast strains during longan fruit wine fermentation at 30 °C in the presence of various LSE concentrations ((**A-1**,**A-2**) 0%; (**B-1**,**B-2**) 0.25%; (**C-1**,**C-2**) 0.5%).

Subsequent properties, such as ethanol conversion in LFW medium supplemented with various concentrations of LSE, are shown in Figure 7. All strains displayed a consistent trend in residual sugar consumption, with levels approximately between 140.15 and 146.05 g/L, and ethanol production ranging from 66.33 to 66.77 g/L at LSE concentrations of 0% and 0.25%, respectively (Figure 7(A-1,A-2)). This can be attributed to the initial conversion of sucrose into glucose and fructose through hydrolysis by invertase, which is located in the cell wall. Subsequently, both glucose and fructose molecules were transported continuously across the cell membrane [41]. On the other hand, a higher concentration of LSE (0.50%) exhibited inhibitory effects on sugar consumption and ethanol production significantly, as shown in Figure 7(C-1,C-2). Among the strains tested, only C6-3 demonstrated complete fermentation, producing 66.64 g/L of ethanol, followed by B5-2 with 61.21 g/L, while SC-5088 yielded the lowest ethanol production at 52.98 g/L. After 9 days of fermentation, residual fructose was detected in the yeast strains B5-2 and SC-5088. This observation can be attributed to the sensitivity of *S. cerevisiae* to LSE. Tannins, now part of LSE, are known to inhibit yeast growth and metabolism during the fermentation process. These inhibitory effects are associated with mechanisms that interfere with membrane-bound reactions, disruption in ion transport in mitochondria [42], and reduction of the rate of oxidative phosphorylation. This is significant given that nearly half of the enzymes in yeast cells can be stimulated or inhibited by phosphorylation [43].

#### 3.4.3. Bioactive Compound, Antioxidant Analysis, and α-Glucosidase Inhibitory Activity

**Figure 8:**
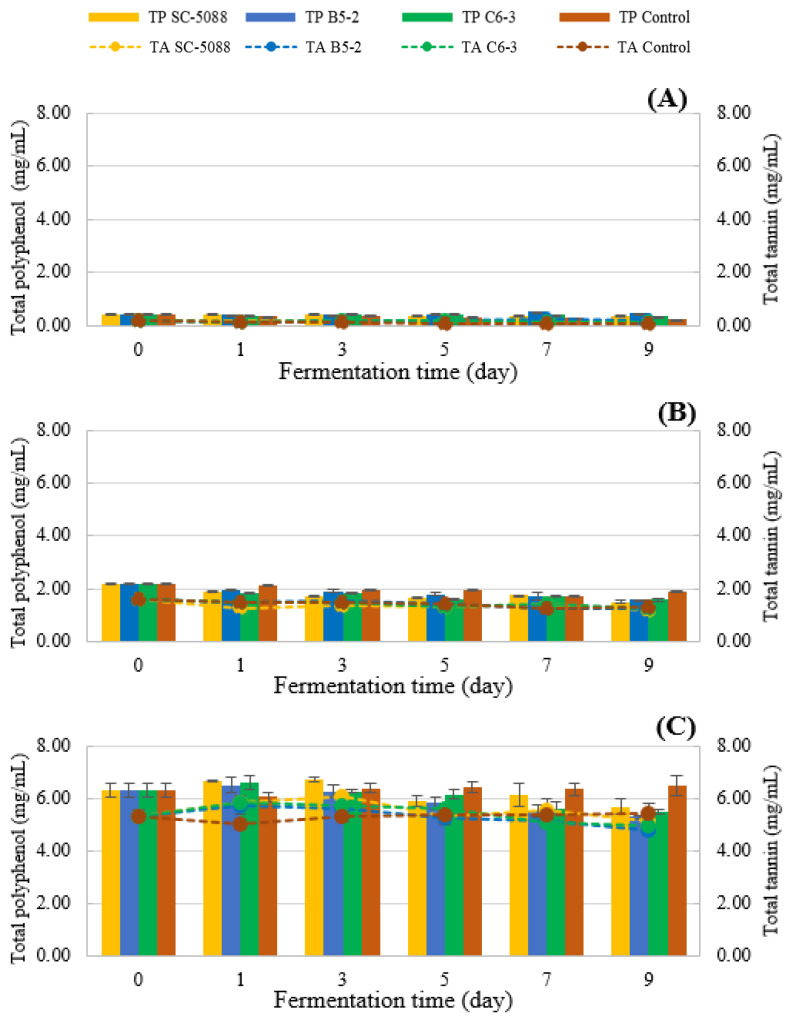
Changes in total polyphenol (bar chart) and total tannin (dash line) during the fermentation of longan fruit wine by selected tannin-tolerant yeasts isolated from Miang compared to the reference strain under the varied LSE concentrations ((**A**) 0%; (**B**) 0.25%; (**C**) 0.5%).

**Figure 9:**
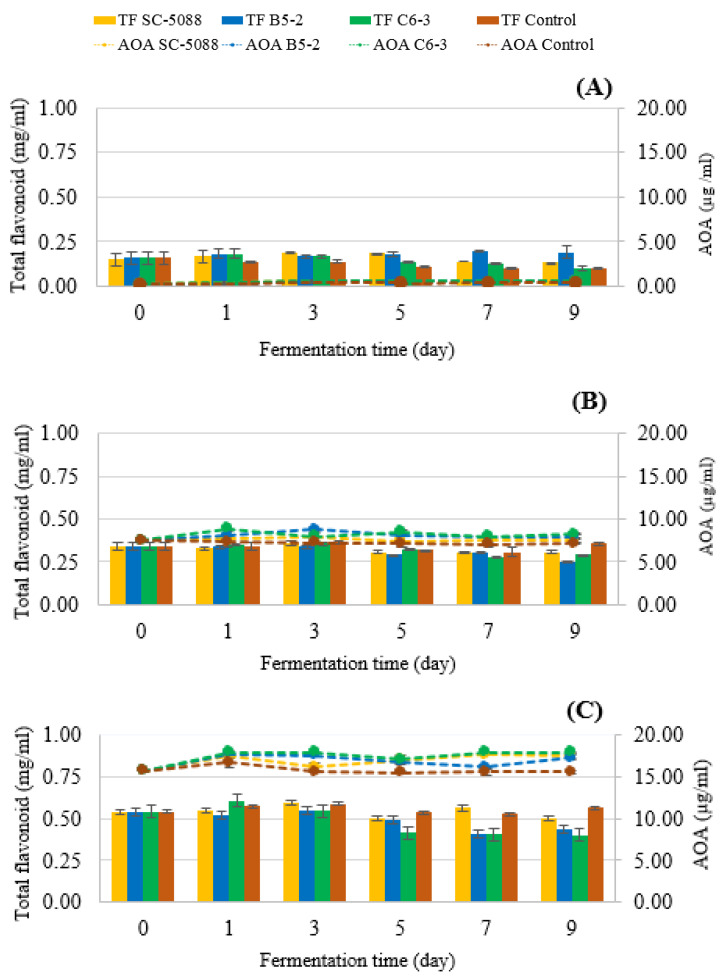
Changes in total flavonoid (bar chart) and AOA (dash line) during fermentation of longan fruit wine supplemented with various LSE concentrations using the selected yeast strains isolated from Miang and the reference strain ((**A**) 0%; (**B**) 0.25%; (**C**) 0.5%).

Overall, there was a decrease in both TP and TT after fermentation, as illustrated in Figure 8. The TP exhibited a reduction of approximately 28–31% and 10–16%, while the TT decreased by about 20–25% and 3–10% across all strains when fermented with LSE at concentrations of 0.25% and 0.50%, respectively. Likewise, TF reduced in the range of 10–26% and 7–25% (Figure 9B,C) of other strains (at LSE 0.25% and 0.50%), respectively. These agreed with a previous study that reported that during alcoholic fermentation, the quantity of TF was the major phenolic compound that decreased after fermentation [44,45]. The decrease in phenolic compounds during fermentation can be attributed to the role played by *S. cerevisiae* in winemaking, as phenolic compounds present in fruit juice are metabolized, converted, or absorbed by yeast cells due to microbial activity [46,47]. Additionally, phenolic compounds may be adsorbed onto yeast cells or precipitated from the solution, further contributing to reduced phenolic levels [48,49]. This phenomenon commonly observed in winemaking has a significant impact on the attributes of wine and the potential health benefits of the final product.

The AOA was assessed in terms of ascorbic acid equivalent (AAE), as presented in Figure 9. At 0% LSE, AOA values for all strains and the control began at 0.28 µg AAE/mL, gradually increasing to a range of 0.38–0.65 µg AAE/mL. With the introduction of 0.25% LSE, AOA values started at a higher level of 7.49 µg AAE/mL, reaching 7.01–8.83 µg AAE/mL. In the case of a higher LSE (0.5%), AOA values also commenced at an elevated level of 15.62 µg AAE/mL, ultimately spanning a range of 15.50–17.94 µg AAE/mL. The presence of LSE (0.25% and 0.5%) significantly boosted AOA values compared to the absence of LSE. Particularly noteworthy was the consistent elevation of AOA values in strains B5-2 and C6-3 when supplemented with LSE, which was much higher than those of the unfermented sample across all concentrations and fermentation days. This observed increase in AOA values during fermentation indicates the active contribution of the fermentation process itself to the development of AOA. These findings highlight the potential of LSE, particularly at higher concentrations, to enhance AOA during the fermentation and processing of LFW. This enhancement is significant for its health-promoting properties. The increased AOA observed in LFW after adding tannin extract and subsequent fermentation implies a structural breakdown of the tannin extract, potentially transformed through microbial hydrolysis, leading to the release or synthesis of various antioxidant compounds [50,51].

The influences of various concentrations of LSE were evaluated for their impact on α-glucosidase enzyme inhibitory activity (Table 4). As the concentration of the extract increased from 0.00% to 0.25% and then to 0.50%, there was a notable increase in inhibition percentages, indicating a clear dose–response relationship. At a concentration of 0.50% LSE, the inhibition reached a substantial level of approximately 95.02%, while at 0.25% LSE, it was notably found at approximately 77.71%. LSE at 0.00% exhibited an inhibition percentage of approximately 28.78%, whereas commercial product B showed about 39.59%. These findings underscore a significant enhancement in α-glucosidase enzyme inhibition with increasing concentrations of LSE. This finding suggests that higher concentrations of LSE are more effective in inhibiting enzyme activity, which indicates potential for managing blood sugar levels linked to hyperglycemia conditions, especially in individuals with early stages of type 2 diabetes. As this potentially slows down the absorption of soluble sugars generated from polymeric carbohydrates such as starch converted into simple sugars, this inhibited stage has the potential to regulate postprandial blood glucose levels and is linked to a reduced risk of hyperglycemia [52,53]. The significant inhibition observed by incorporating LSE into the diet may support controlling blood sugar spikes and glycemic conditions after meals, thereby contributing to overall metabolic health. Additionally, considering its potent α-glucosidase inhibitory activity and antioxidant properties, LSE holds potential as a valuable ingredient in functional foods and nutraceuticals targeted towards individuals with early stages of type 2 diabetes or those aiming to manage their blood sugar levels coupled with better diets. Longan fruit and its functional metabolites offer medicinal benefits and are traditionally used in Chinese herbal medicine. Longan seeds have indicated therapeutic uses in treating various conditions [25,54]. Modern research highlights the potential of longan as a superfruit and its extracts for food and pharmaceutical applications [55]. Recent studies have explored innovative applications of LSE, including its incorporation into bead-forming drug delivery systems, highlighting its versatility in modern pharmaceutical technology [56]. Despite the lack of direct reports on the utilization of LSE in food products, its extensive use in traditional medicine and ongoing research into its pharmacological properties suggest its potential as a valuable dietary supplement or potential functional ingredient.

The comparison involving LFW supplemented with varying levels of LSE (0%, 0.25%, and 0.50%), using the *S. cerevisiae* C6-3, and commercial longan fruit products labeled as A and B is shown in Table 4. Our findings indicate that the addition of LSE at 0.25% and 0.5% to LFW led to a substantial increase in TP, nearly 6 and 19 times higher, respectively, compared to commercial product B, which had a TP of 0.29 g/L. Similarly, the AOA showed a remarkable increase of approximately 31 and 70 times higher, respectively, compared to that of commercial product B.

**Figure 10:**
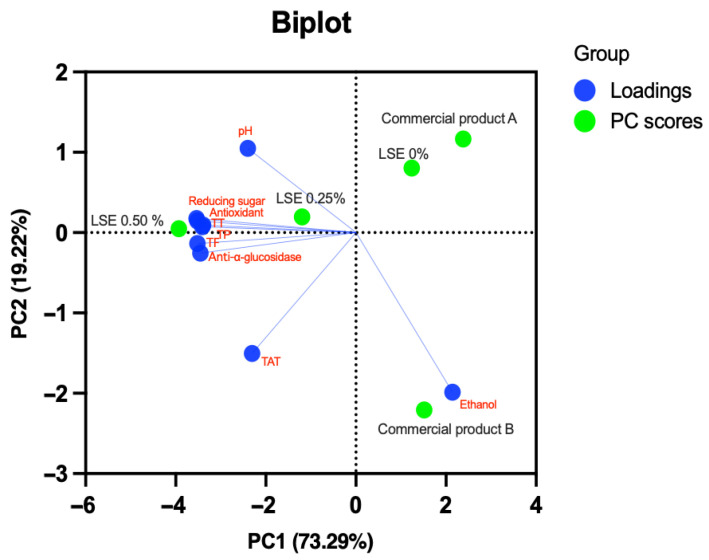
PCA biplot of pH, TAT (total acid titration), reducing sugar, ethanol, bioactive compounds, and antioxidant activity in longan fruit wine supplemented with varied levels of LSE, compared to commercial products.

A PCA was employed to characterize the parameters of LFW products, including pH, total acid titration (TAT), reducing sugar, ethanol, and bioactive compounds (TP, TT, and TF), AOA, and anti-alpha-glucosidase activity, which are presented in Figure 10. The PCA results revealed a total variance of 92.51, with PC1 accounting for a substantial proportion (73.29%) and PC2 contributing an additional 19.22%. The strong dominance of PC1 suggests that it encapsulates the primary sources of variability among the studied parameters. Interestingly, positive associations were discerned between LSE 0% and commercial product A, indicating similarities in their compositional profiles. However, as the concentration of LSE increased (0.25–0.50%), a notable augmentation was observed in the levels of bioactive compounds, specifically TP, TT, TF, antioxidant, and alpha-glucosidase inhibitory activity. This indicates that the introduction of LSE at these concentrations positively influences the content of these bioactive components in LFW. Furthermore, examining the loading scores on PC1 and PC2 provides insights into the variables contributing most significantly to the observed variations. For instance, ethanol exhibited a moderate positive loading on PC1, while bioactive compounds (TP, TT, TF, antioxidant, and alpha-glucosidase inhibitory activity) displayed substantial negative loadings. On PC2, ethanol again featured prominently with a very large negative loading, distinguishing its impact on the secondary axis of variability.

The LFW products fermented by tannin-tolerant *S. cerevisiae* displayed significant differences when supplemented with LSE at levels of 0.25% and 0.50%. The addition of LSE offered an alternative approach to enhancing the health benefits of this fruit wine product. This enhancement was also correspondingly achieved through the increased presence of beneficial compounds such as phenolic compounds with associated AOA. LSE was previously known for its potent anti-cancer potential [54], as well as its anti-diabetic and anti-hyperglycemic properties [53]. Therefore, LFW products analyzed and advanced from this research provide a strong foundation for promoting LFW-supplemented seed extract as a functional health beverage option.

## 4. Conclusions

This study successfully isolated tannin-tolerant yeasts capable of ethanol production from a traditional fermented tea leaf product called Miang collected in Laos. The phenotypic characteristics and molecular identification through sequence analysis of the D1/D2 region confirmed those strains to be *Saccharomyces cerevisiae*, *Candida tropicalis,* and *Kazachstania humilis*. The isolated *S. cerevisiae* strains exhibited greater fermentation efficacy when compared to the well-recognized reference strain upon cultivation in a tannic acid-enriched medium. Furthermore, the tannin-tolerant *S. cerevisiae* strains demonstrated significant potential in the fermentation of LFW with the supplementation of LSE and showed the benefits of value-added applications in tannin-rich substrate fermentations, potentially enhancing the presence of health-relevant compounds, boosting AOA, inhibiting α-glucosidase activity, and contributing to targeted health benefits.

## Figures and Tables

**Table 1:** Miang sampling sites and the number of yeast isolates from different locations.

No.	Regions	No. of Samples	No. of Yeast Isolate	No. of Gas Formation	No. of Ethanol Producing Yeast	Location
1	Xayaboury district	9	7	2	0	19.268576″ N, 101.707712″ E
2	Xienghorn district	15	13	4	3	19.587956″ N, 100.807902″ E
3	Khop district	9	15	4	3	19.41′03.2″ N, 100.30′07.1″ E
4	Saysathan district	10	0	0	0	19.405888″ N, 101.387887″ E
	Total	43	35	10	6	

**Table 2:** Molecular identification of ethanol-producing yeast using D1/D2 sequence analysis.

Isolate (Code)	Species Name	Similarity (%)	Query Length (bps)	Accession Number
B3-2	*Kazachstania humilis*	100	605	KY106507.1
B5-1	*Saccharomyces cerevisiae*	100	603	NG_042623.1
B5-2	*Saccharomyces cerevisiae*	100	583	NG_042623.1
C4-6	*Candida tropicalis*	100	594	KU729147.1
C6-3	*Saccharomyces cerevisiae*	99.64	596	NG_042623.1
C6-10	*Candida tropicalis*	100	600	KU729147.1

**Table 3:** Ethanol fermentation in varied concentrations of tannic acid for 24 h at 30 °C.

Parameter	TA Conc. (%)	*S. cerevisiae* B5-1	*S. cerevisiae* B5-2	*S. cerevisiae* C6-3	*S. cerevisiae* 5088
Ethanol Productivity (g/L·h) at 24 h	0.00	0.37 ± 0.01	0.37 ± 0.01	0.37 ± 0.01	0.37 ± 0.01
	1.00	0.29 + 0.01 ^b^	0.36 ± 0.01 ^a^	0.36 ± 0.01 ^a^	0.35 ± 0.01 ^a^
	2.50	-	0.23 ± 0.01 ^a^	0.21 ± 0.01 ^b^	-
	5.00	-	-	-	-
Ethanol fermentation efficiency (%) at 24 h	0.00	87.31 ± 0.21	87.43 ± 0.98	86.80 ± 0.31	87.86 ± 1.87
	1.00	74.81 ± 1.77 ^b^	83.79±1.54 ^a^	84.39 ± 0.29 ^a^	83.75 ± 0.81 ^a^
	2.50	-	54.03 ± 0.88 ^a^	49.92 ± 0.76 ^b^	-
	5.00	-	-	-	-

Note: means in rows with different superscripts are statistically different at *p* < 0.05.

**Table 4:** The component of longan fruit wine supplemented with longan seed extract and commercial products.

Parameters	LSE 0%	LSE 0.25%	LSE 0.50%	Commercial Product A	Commercial Product B
pH	4.49 ± 0.01 ^a^	4.46 ± 0.01 ^a^	4.48 ± 0.01 ^a^	3.52 ± 0.01 ^b^	3.37 ± 0.01 ^b^
TAT (g/L)	0.05 ± 0.01 ^b^	0.07 ± 0.01 ^ab^	0.08 ± 0.01 ^a^	0.03 ± 0.01 ^c^	0.08 ± 0.01 ^a^
Reducing sugar (g/L)	1.61 ± 0.03 ^c^	2.97 ± 0.16 ^b^	4.34 ± 0.24 ^a^	0.63 ± 0.01 ^d^	1.00 ± 0.10 ^d^
Ethanol (% *w*/*v*)	6.65 ± 0.72 ^b^	6.61 ± 0.17 ^b^	6.64 ± 0.9 ^b^	5.45 ± 0.13 ^bc^	10.81 ± 0.28 ^a^
TP (g/L)	0.33 ± 0.01 ^c^	1.59 ± 0.05 ^b^	5.49 ± 0.09 ^a^	0.15 ± 0.02 ^c^	0.29 ± 0.30 ^c^
TT (g/L)	0.12 ± 0.02 ^c^	1.27 ± 0.05 ^b^	4.97 ± 0.11 ^a^	0.03 ± 0.01 ^c^	0.07 ± 0.22 ^c^
TF (g/L)	0.10 ± 0.01 ^c^	0.29 ± 0.01 ^b^	0.40 ± 0.04 ^a^	0.07 ± 0.01 ^c^	0.13 ± 0.01 ^c^
Antioxidant (µg/mL)	0.59 ± 0.01 ^c^	8.25 ± 0.39 ^b^	17.88 ± 0.13 ^a^	0.14 ± 0.01 ^c^	0.26 ± 0.02 ^c^
Anti α-glucosidase (%)	28.78 ± 2.12 ^d^	77.71 ±0.03 ^b^	95.02 ±0.44 ^a^	19.90 ± 0.58 ^e^	39.59 ± 0.82 ^c^

Note: means in rows with different superscripts are statistically different at *p* < 0.05.

## Data Availability

The original contributions presented in the study are included in the article, further inquiries can be directed to the corresponding author.
